# Comparing cystic fibrosis outcomes across the pond

**DOI:** 10.1136/thoraxjnl-2014-206393

**Published:** 2014-12-23

**Authors:** David C Taylor-Robinson, Michael S Schechter, Rosalind L Smyth

**Affiliations:** 1 Department of Public Health and Policy, University of Liverpool, Liverpool, UK; 2 UCL Institute of Child Health, London, UK; 3 Department of Pediatrics, Division of Pulmonary Medicine, Virginia Commonwealth University, Children's Hospital of Richmond at VCU, Richmond, Virginia, USA

**Keywords:** Cystic Fibrosis, Paediatric Lung Disaese, Clinical Epidemiology

Analysis of registry data has provided key insights into the changing demographics, outcomes and treatments in cystic fibrosis (CF), but few studies have made use of the great potential for cross-country comparisons. The linked study by Goss and colleagues^1^ in *Thorax* does just this, using registry data collected in the UK and the USA to compare CF outcomes and use of treatments on opposite sides of the Atlantic. The study certainly makes for uncomfortable reading from a UK perspective, but raises more questions than it addresses. The analysis suggests that the USA does better in terms of lung function in children, and the authors conclude this is due to more intensive treatment in the early years. But is this interpretation correct?

One of the most important findings of the study is the striking gap in mean % predicted FEV_1_ between the UK and the USA at around 6 years, the age at which lung function can be consistently collected and measured reliably at all care centres. The gap subsequently narrows and disappears by age 30. Overall, children under 12 years of age in the UK had significantly lower lung function by 7.6 percentage points compared with children in the USA. This is a big difference in a study where the large sample sizes mean that the estimations of population level differences are quite precise, even for imprecisely measured outcomes such as % predicted FEV_1_.^2^

In these initial attempts to compare data collected in slightly different ways in different countries, it is important to ensure that the comparisons are valid. For example, we must ensure that the findings of this analysis are not biased by the possibility that the samples used in the study are not fully representative of the entire CF populations in their respective countries. Ascertainment bias is a well-recognised problem in comparative registry studies, and a key question with regard to the study by Goss *et al* is the extent to which the two populations can be fairly compared. Comparative analysis of coverage data in CF registries has demonstrated how this can influence outcomes when registries are differentially representative of their respective total estimated population.^3^

In the UK, the registry is estimated to capture almost all of the CF population, and any patients attending the National Health Service clinics will have data routinely collected into the database.^4^ By contrast, the US registry collects data on patients seen in US accredited CF centres and may have lower coverage.^5^ Older estimates suggest that coverage in the US registry was about 75% in the 1990s,^3^ during a period when the UK CF survey, a precursor of the UK registry, had almost complete coverage of the UK population.^6^ It is possible that the US registry does not capture individuals across the full range of the socio-economic spectrum to the same extent as the UK registry due to the nature of the healthcare system in the USA, where the uninsured and those with poorer access to care may be under-represented. Thus, a consequence of the high level of population coverage in the UK, coupled with a universal healthcare system, is that the UK registry may more accurately represent more disadvantaged groups, which we know from studies in the UK and the USA have worse clinical outcomes.^4^ ^7^

Let us assume that the US dataset does not represent the most disadvantaged to the same extent: could this create the differences observed? In order to explore this further, we repeated the analysis in the paper by Goss *et al*, removing 20% of patients from the most disadvantaged areas in the UK from the analysis. This reduced the gap between the UK and the USA, but not by much, suggesting that ascertainment bias may potentially explain some, but not most, of the difference in childhood lung function. Concerns about ascertainment aside, a key strength of the study by Goss *et al* is the hard work that has gone into harmonising the datasets to ensure comparability, and the extent to which the authors have tested the robustness of their findings by stratifying the analyses and undertaking appropriate adjustment for important confounders in their statistical models. For example, it is plausible that differences in lung function could arise due to variance in the ethnic and genetic makeup of the US and UK populations, but the authors address this by restricting the analysis to white patients, and also by repeating the analysis using only patients who are homozygous for F508del mutation. All the analyses show similar results.

Understanding the narrowing of the US–UK gap with increasing age is challenging due to the cross-sectional nature of the study, which is an important limitation. We should not overinterpret the age related trends in the figures since these do not accurately represent longitudinal lung function decline in the populations over time. These cross-section differences conflate cohort effects, and survivor bias, whereby the cross-sectional comparisons at older ages represent only the healthier individuals who have survived to the point of analysis. Without understanding any survival differences, these patterns are very hard to interpret. This brings us back to the significance of the early difference in lung function, which represents the ‘cleanest’ comparison of the data, since very few patients die in the first few years of life and so survivor bias does not complicate the picture.

Overall, it seems most likely that the UK–US gap in early lung function gap is real, even if it may not be quite as great as stated. So, how can we explain this difference? The authors suggest earlier and more aggressive use of chronic pulmonary therapies may be the reason, given the large discrepancies in prescribing patterns evident in the use of both nebulised saline and DNase: children in the USA are much more likely to receive these therapies. However, there is no direct evidence presented to support this conclusion, and it is a potentially dangerous leap of faith to suggest that we should be systematically delivering therapies to very young children in the absence of good evidence of effectiveness.

The authors’ conclusions will likely fuel the debate around the effectiveness of early intensive intervention in preschool children with CF on the basis that this may postpone or prevent early lung disease^8^ but data informing the discussion remain scant. A Cochrane review shows short and intermediate term improvements in lung function from DNase therapy, as well as reductions in pulmonary exacerbations, supporting its use in the general CF population, but a subgroup analysis focusing on preschool children was not possible given the dearth of data in this age group.^9^ Inhaled hypertonic saline, by contrast, has been shown to improve quality of life and reduce pulmonary exacerbations, but does not appear to have a substantive effect on lung function.^10^ A recent study of hypertonic saline in children under 6 years of age failed to show a difference in pulmonary exacerbations, the primary study endpoint. Infant pulmonary function testing performed as an exploratory outcome in a subgroup did not demonstrate significant differences between groups except for a small mean improvement in forced expiratory volume in 0.5 s in the active treatment group.^11^

Plausible alternatives to explain the lung function gap could relate to organisation of care, particularly frequency of access to centre-based care. Children attending large centres more frequently in the early years in the UK do better,^12^ and a challenge to services in the UK is to achieve universally high standards of care across the whole country. Data from the US also suggest that the centres with highest lung function scores for their patients were characterised by more clinic visits, more respiratory tract cultures and frequent antibiotic treatment of patients, particularly those considered to have mild lung disease.^13^ ^14^ Optimum treatment of pulmonary exacerbations is a key factor in terms of achieving better pulmonary outcomes,^15^ and this may further explain some of the differences seen in the study, but these data are not comparable across registries at the moment.

Clearly, more trials are needed in younger children before clear conclusions may be drawn regarding how superior outcomes may be obtained in this age group. While we can do more to unravel the causal association between use of therapies and long-term outcomes using registry data, this requires longitudinal studies and modern statistical approaches to better establish causal pathways and eliminate the problem of confounding by indication in observational data.^16^

We welcome the study by Goss *et al* and the questions that is raises. Harnessing the rich data in CF registries offers the opportunity to improve the lives of patients with CF, and cross-country comparisons have changed policies and practice in CF in the past.^17^ Just as centre-based comparisons within countries have increased interest in benchmarking and quality improvement initiatives in an attempt to drive up standards,^18–20^ we hope that further cross-national comparisons such as the one presented here can be used to highlight potentially important differences in outcomes and care for people with CF between countries.
